# Frequency of anger and its potential relationship with Selfesteem and Adverse Childhood Experiences among Medical and Sociology undergraduate students in Pakistan

**DOI:** 10.12669/pjms.39.2.6113

**Published:** 2023

**Authors:** Farrukh Ansar, Hira Naveed, Almas Khattak, Sylvia Ali Khan

**Affiliations:** 1 Farrukh Ansar, MBBS., Northwest School of Medicine, Khyber Medical University Peshawar, Pakistan; 2 Hira Naveed, BS Sociology, Higher Education Commission, Islamabad, Pakistan; 3 Almas Khattak, MBBS, MPH, GDCE, Northwest School of Medicine, Khyber Medical University Peshawar, Pakistan; 4 Sylvia Ali Khan, MBBS, MRC(Psych), Northwest Teaching Hospital, Peshawar, Pakistan

**Keywords:** Clinical anger, Self-esteem, Adverse childhood experiences, Medical students

## Abstract

**Objectives:**

To determine the frequency of clinical anger, adverse childhood experiences, self-esteem, and their mutual relationship among undergraduate medical and sociology students.

**Methods:**

In this descriptive cross-sectional study, data from 400 undergraduate medical and sociology students was collected from the Northwest School of Medicine, Peshawar, and the sociology department of the International Islamic University, Islamabad, from July to September 2021. A self-administered questionnaire incorporating a Clinical Anger Scale (CAS), Adverse childhood experience (ACE) scale and Rosenberg self-esteem (RSE) scale was utilized for data collection. Descriptive statistics, ANOVA and logistic regression model were executed to perform data analysis using SPSS.

**Result:**

The mean CAS score was 19.65 ± 13.23 suggesting that 60.2% of the participants experienced mild to severe degrees of clinical anger. Females were experiencing more anger issues than males (64% vs 54%, p = .040) RSE scale showed that 72.8% of the participants had low self-esteem with a mean score of 12.70 ± 5.43. Besides, 51.5% of the participants had at least one type of adverse childhood experience while 15.8% of the sample population reported being sexually abused in childhood. Significant correlation was found between CAS and age, ACEs and RSE score (p = <0.01). The logistic regression model also suggested that the prevalence of anger was higher in individuals with ACEs (OR = 1.29, 95% CI: 1.12, 1.14, p = <.001) and low self-esteem (OR = 1.15, 95% CI: 1.09, 1.22, p = <.001).

**Conclusion:**

The high frequency of clinical anger necessitates the implementation of periodic screening across all universities. Keeping in mind the problem of low self-esteem and the history of ACEs, there is an urgent need for the development of strategies to preserve and improve the mental well-being of the young generation.

## INTRODUCTION

Emotions are an imperative part of human behavior as they are essential to endured existence of the humans’ race but it is also evident that emotional distress can lead to psychological, social, and physical illnesses.^1^ Anger; one of the crucial human emotions plays a pivotal role in a person’s balanced emotional health as it can enact positive change by conveying upsetting social situations.^2^ The need for anger in the social life of human beings is minimal, excessive and frequent manifestation of anger can have catastrophic effects on overall mental and physical well-being.^3^ Studies have suggested that prolonged anger episodes of anger alter the normal neurochemical and hormonal processes which subsequently results in an increased risk of developing coronary heart diseases, diabetes, stroke, hypertension, gastric and endocrine disorders.^4^ Anger can also adversely affect the mental health of an individual by impairing cognition, semantic decision-making and behavioral processes.^5^

Anger is influenced by a large variety of factors, including current lifestyle, past experiences, especially adverse childhood events like physical, emotional, and sexual abuse.^6^ Such traumatic experiences are precursors of stress, frustration, cognitive impairment, and decreased self-esteem that led to anger and aggressive behavior.^7^ Historical literature has shown that the etiology of abnormal anger levels is multifactorial such as low self-esteem, depression, obsessive-compulsive disorder, oppositional defiant disorder, post-traumatic stress disorder anxiety and routine stress can significantly increases the chances of aggressive behavior.^8^,^9^ Research has also shown that Adverse Childhood Experiences (ACEs) depression and anxiety can be considered as precursors of clinical anger.^10^,^11^ Unfortunately, literature shows that the prevalence of anxiety and depression is quite high among Pakistani university students.^12^,^13^ However, there is no credible data showing the burden of anger among university students as these undergraduates will occupy important positions in health care and social services sector in near future.

The purpose of the present study was to find out the prevalence of anger, ACEs, and levels of self-esteem among medical students and in university students of other disciplines and to uncover any potential relationship between anger, self-esteem, and ACEs that are considered as predictors of aggression and clinical anger.

## METHODS

This descriptive cross-sectional study was conducted among students of Northwest School of Medicine, Peshawar and the sociology department of International Islamic University, Islamabad from July to September 2021. The ethical approval was granted by the Institutional Review and Ethics Board (IREB) of Northwest School of Medicine, Peshawar. (Ref No: IREB/EAC/006) Students aged above 18 years and who gave consent to participate in the study were included in the study. Sample size was calculated using OpenEpi, keeping the population size one thousand, anticipated frequency 50%, margin of error 5% and design effect one at 97% confidence level, the calculated sample size was 400. Convenience sampling technique was used and 200 participants were recruited from each discipline, i.e., medicine and sociology. Data was collected using the google Forms platform, questionnaires were uploaded to the digital form that incorporates an informed consent form, instructions regarding the questionnaire and important information about study objectives. The google forms were circulated among students through class WhatsApp groups with the help of student affairs’ representatives, who were directed by the administration to help with data collection.

Our study instrument had five main sections. The first and second sections comprised the consent statement and baseline characteristics. While the third, fourth and fifth sections were comprised of internationally validated Clinical Anger Scale (CAS), Rosenberg Self-Esteem (RSE) scale and Adverse Childhood Experiences (ACEs) scale. The CAS scale consisted of 21 questions with four different options for each statement. Each question was then assigned a score on a 4-point Likert scale, with options A = 0, B = 1, C = 2, and D = 3. According to developer’s guidelines, CAS score was interpreted as; score from 0-13 - “minimal clinical anger” 14-19 - “mild clinical anger”, 20-28 - “moderate clinical anger” and 29-63 - “severe clinical anger”. The RSE sale consists of ten questions; respondents rate the extent to which they agree with each statement on a four-point Likert scale with options, “Strongly Agree”, “Agree”, “Disagree” and “Strongly Disagree” scored as 3, 2 and 1 point respectively. However, questions 2, 5, 6, 8, 9 were reverse-scored to control any potential bias. A total score is calculated by adding the score for all responses which may range from 0 to 30, with greater scores indicating higher self-esteem. The ACEs questionnaire has 10 items designed to assess ten types of traumatic childhood experiences including; verbal, emotional, physical, and sexual abuse. Each item has two options “No” and “Yes” which are scored as 0 or One point respectively.

IBM® SPSS® Statistics version 23.0 was utilized for data entry and analysis. The analysis was performed at 95% Confidence intervals with a significance level set at 0.05. The numeric and string properties were adjusted for variables and descriptive statistics were utilized for calculating frequencies, means and their standard deviation. Differences in mean scores of different groups were calculated through one-way Analysis of Variance (ANOVA). A logistic regression analysis was executed with the dichotomized clinical anger as the dependent variable. A correlation analysis was also performed to find out any potential relationship between CAS, RSE and ACEs score.

## RESULTS

A total of 400 responses were included in final analyses, of whom 227(56.8%) were male and 173(43.3%) were female participants. The mean age was 22.2 ± 2.69 years (range: 17 to 30 years). The sample included 200 undergraduate students from Faculty of Medicine and 200 students from Faculty of Social Sciences. Overall, 56 students were from first year, 71 from second year, 70 from third year, 147 and 56 from final year respectively. Baseline characteristics of participants is shown in Table-I.

**Table-I:** Baseline characteristics of participants (N = 400).

Characteristic	Category	Frequency (%)
Gender	Male	227(56.8%)
	Female	173(43.3%)
Age (Mean ± SD)		22.2 ± 2.69 years
Smoker	Yes	61 (15.3%)
	No	339 (84.8%)
Alcoholic	Yes	6 (1.5%)
	No	394 (98.5%)
Vegetables and fruits in daily diet	Yes	122 (30.5%)
	No	278 (69.5%)
Strenuous physical activity per week	<75 min	114 (28.5%)
	>75 min	206 (51.5%)
	Nil	80 (20%)
Sleep duration per 24 hours	> 6 hours	56 (6.5%)
	6 to 8 hours	321 (80.2%)
	< 8 hours	53 (13.3%)
BMI	Below 18.5	16 (4%)
	18.5 – 24.9	360 (90%)
	25.0 – 29.9	24 (6%)

The mean CAS score was found to be 19.65 ± 13.23. As per scoring criteria of CAS 159 (39.8%) participants fall in “minimal clinical anger” category, 42 (10.5%) students were in “mild clinical anger” category while 85 (21.3%) and 114 (28.5%) students were classified in “moderate clinical anger” and “severe clinical anger” categories respectively. CASS score significantly increased with higher level of class. The mean CASS score in first, second, third and fourth year were 13.02 ± 12.86, 18.66 ± 11.74, 22.23 ± 12.73 and 23.14 ± 13.40 respectively. (p-value=>.001) Score comparison on basis of gender, field of study and smoking status is shown in Table-II. Overall, 54% of male population had anger issues up to some extent while 64% of the females were experiencing clinical anger (p-value = 0.040). Clinical anger was significantly higher in social sciences students than medical students having a prevalence of 65% and 53% in sample population (p-value = 0.025). Additionally, correlation analysis reported that CAS was significantly correlated with age, RSE and ACE score with r = 0.201, r = 0.244 and r = 0.443 respectively (p-value = < 0.01).

**Table-II:** Score comparison of various scores among demographic characteristics (N = 400).

Scale	Characteristic	Mean (SD)	Std. Error	95% CI for Mean		F	P value

				Upper	Lower		
CAS Score	Male	18.75 (13.1)	.875	17.03	2.480	2.381	0.124
	Female	20.82 (13.2)	1.00	18.83	22.80		
	Medical students	17.89 (12.4)	.880	16.15	19.63	7.168	0.008
	Sociology Students	21.41 (13.7)	.974	19.48	23.33		
	Smokers	22.84 (16.8)	2.15	18.52	27.15	4.215	0.041
	Non-smokers	19.07 (12.4)	.674	17.75	20.40		
RSE Score	Male	12.90 (5.1)	.334	12.24	13.55	0.719	0.397
	Female	12.43 (5.9)	.450	11.54	13.32		
	Medical students	12.73 (5.7)	.405	11.93	13.52	0.010	0.920
	Sociology Students	12.67 (5.1)	.363	11.95	13.39		
	Smokers	14.56 (5.5)	.714	13.13	15.99	8.589	0.004
	Non-smokers	12.36 (5.3)	.291	11.79	12.93		
ACE Score	Male	1.52 (1.9)	1.32	1.25	1.781	2.478	0.116
	Female	1.23 (1.5)	1.24	0.98	1.472		
	Medical students	1.55 (1.8)	.129	1.29	1.801	2.710	0.100
	Sociology Students	1.24 (1.8)	.133	0.98	1.500		
	Smokers	2.39 (2.2)	.293	1.81	2.981	22.02	>.001
	Non-smokers	1.21 (1.7)	.093	1.03	1.402		

RSE scale scores estimated that 291 (72.8%) students had a score of less than 15 which suggests low self-esteem. 99 (24.8%) students scored in between 15-25 which can be interpreted as normal self-esteem, whereas 10 (2.5%) individuals showed a high self-esteem. The mean RSE scale score calculated was 12.70 ± 5.43.

Present study also found that according ACE score, 206 (51.5%) participants had an experience at least one type (maybe multiple times) of childhood trauma or abuse also known as ACEs. Out of exposed individuals, 154 (74.75%) experienced 1-3 types of ACEs while 42 (20.38%) individuals had an experience of 4-6 types of ACEs whereas 10 (4.85%) participants experienced 7 or more types of ACEs. The most common type of ACE was related to emotional abuse by a parent of household elder which was experienced by 109 (27.25%) out of total sample population. The second most common ACE was physical abuse by a parent or other adult in household which was reported by 68 (17%) individuals. Third most frequent and concerning ACE was sexual abuse, 63 (15.8%) participants including 34 males and 29 females answered “Yes” to the question “Did an adult or person at least five years older than you ever touch or fondle you or have you touch their body in a sexual way or attempt or actually have oral, anal, or vaginal intercourse with you?” Table-III shows logistic regression analysis which concluded that clinical anger was significantly associated with gender, field of study, RSE and ACE scores.

**Table-III:** Relationships between clinical anger and baseline characteristics of participants (N = 400).

Variables	B	S. E	Wald	P value	OR	95% CI for OR	

						Upper	Lower
Gender	-.807	.288	7.871	.005	0.446	0.254	0.784
Study discipline	-.767	.270	8.093	.004	0.464	0.274	0.788
Smoker	.366	.368	0.992	.319	1.442	0.702	2.965
Physical Activity	-.216	.398	0.295	.587	0.805	0.369	1.758
RSE Score	.261	.029	24.57	<.001	1.157	1.092	1.226
ACE Score	.146	.072	13.01	<.001	1.298	1.126	1.495
Constant	3.42	.701	23.80	<.001	.033		

## DISCUSSION

The current study attempted to determine the prevalence of clinical anger among undergraduate students and to investigate any potential relationship between clinical anger and factors like self-esteem, traumatic childhood experiences and other sociodemographic characteristics. Present results demonstrated that 60% of the sample population was suffering from clinical anger (mild to a severe degree). A survey from a rural district of India also reported that 61.7% of the adolescents in the study sample were diagnosed with aggression (clinical anger).^12^ Similar results were yielded from a study conducted in Punjab, which reported that the prevalence of aggression was 51.9% among youngsters.^14^

Numerous high-profile studies have shown robust proof that being an undergraduate university student may act as a significant factor in developing anxiety and stress which is a pivotal public health problem and exposes the students to a higher risk of physical, mental, and emotional issues including uncontrolled anger.^15^ This fact may be attributed to the increased number of participants experiencing a higher degree of anger in our study because of potentially increased levels of stress. Current study observed that the mean CAS score was higher in female students than males suggesting a higher prevalence of clinical anger among females but the results were statistically non-significant.

A Malaysian study reported that men are more likely to suppress their emotions, but their internal anger is significantly higher than their counterpart gender.^16^ However, few studies reported that males were more often affected by clinical anger than females.^17^,^18^ Results also showed that the mean anger score increased in the higher study years, there was a significant difference in the CAS score of first and fourth-year students. This might be incited due to raised stress among students of higher classes because of difficult studies and the burden of other responsibilities that may have impacted the emotional health of students’ leading to thrust in anger. This hypothesis can be supported with evidence provided by a Malaysian study that concludes that stress among university students of upper years was significantly higher than in junior years.^19^

Our results have shown that those who had one or more ACE were prone to developing anger issues than those who didn’t have ACEs. The results from a large sample study led by American researchers are also in parallel with our findings, showing a strong positive relationship between ACEs and anger.^20^

Additionally, our results showed that low self-esteem correlated with inclined anger scores. Results from a 7-yearlong study bolster our evidence by concluding that increased self-esteem was associated with the decline in anger and aggressive behavior.^21^ Similar outcomes were shown by a Korean study stating, self-esteem was negatively associated with anger expression which explains that high self-esteem is helpful in the management of inappropriate anger.^22^ A high-quality meta-analysis also suggested a theoretical model that favors the evidence of association between low self-esteem and aggression.^23^ Researchers have demonstrated that low self-esteem is the root-cause of active and passive aggression. Individuals with low self-esteem sometimes use anger as a defense mechanism when they sense their opinions and feelings don’t mean much to others.^2^

The current study is the first and only one of its kind in Pakistan, it discovered not only the prevalence of clinical anger, low self-esteem, and adverse childhood experiences in the undergraduate population but also their potential correlation. General practitioners and psychiatrists should use screening tools to consider clinical anger, low self-esteem, and adverse childhood experiences as the root causes of a variety of illnesses and symptomatic disorders when making differential diagnoses in young patients with psychiatric symptoms. It is also critical for universities and policymakers to comprehend these reasons among disturbed and less-performing students and offer them timely support. The development of reliable screening tools and their use for screening at regular intervals would prevent serious psychiatric disorders and lower the burden on the health sector.

### Limitations of the study:

First, the sample size is not representative of all universities and study disciplines. Second, the cross-sectional design of the study is insufficient to find firm associations between anger and associated factors. The third problem could be the sampling strategy as there is a sufficient chance that participants may under-rated their true feelings due to self-administered nature of questionnaire. However, the present study sought to identify the burden of anger clinical which requires the immediate attention of authorities and policymakers to take necessary measures in time to prevent further damage to mental health of youth. Moreover, the different factors contributing to anger issues, especially in undergraduate students, shall be explored in more detail through exploratory and qualitative studies.

## CONCLUSION

Current findings have depicted that Anger is common in university students which might have devasting consequences for the mental and physical health of youth. Clinical anger has shown a significant relationship with low self-esteem and especially Adverse childhood experiences, which is a matter of concern and requires immediate attention of healthcare givers and policy makers to devise strategies for the prevention of these adverse events. Besides, there is a grave need for the development of protocols and instruments for early detection and effective interventions for clinical anger which will benefit a large segment of the general population of all age groups.

### Authors’ Contribution:

**FA:** Conceptualized, designed, data collection, statistical analysis, manuscript writing, revision, final approval of manuscript and is responsible for integrity of research.

**HN:** Data collection, data entry, statistical analysis, manuscript writing and editing, proof reading and revision.

**AK & SK:** Data collection, manuscript writing, editing of manuscript, proof reading and revision.

All authors provided critical feedback and helped shape the research, analysis, and manuscript.
